# Therapeutic potential of CRISPR/Cas9 gene editing in engineered T‐cell therapy

**DOI:** 10.1002/cam4.2257

**Published:** 2019-06-14

**Authors:** Qianqian Gao, Xuan Dong, Qumiao Xu, Linnan Zhu, Fei Wang, Yong Hou, Cheng‐chi Chao

**Affiliations:** ^1^ BGI‐Shenzhen, Beishan Industrial Zone Shenzhen China; ^2^ Shenzhen Key Laboratory of Genomics Beishan Industrial Zone Shenzhen China; ^3^ Guangdong Enterprise Key Laboratory of Human Disease Genomics Beishan Industrial Zone Shenzhen China; ^4^ BGI Education Center University of Chinese Academy of Sciences, Beishan Industrial Zone Shenzhen China; ^5^ AbVision, Inc Milpitas California

**Keywords:** CRISPR, gene editing, immunotherapy

## Abstract

Cancer patients have been treated with various types of therapies, including conventional strategies like chemo‐, radio‐, and targeted therapy, as well as immunotherapy like checkpoint inhibitors, vaccine and cell therapy etc. Among the therapeutic alternatives, T‐cell therapy like CAR‐T (Chimeric Antigen Receptor Engineered T cell) and TCR‐T (T Cell Receptor Engineered T cell), has emerged as the most promising therapeutics due to its impressive clinical efficacy. However, there are many challenges and obstacles, such as immunosuppressive tumor microenvironment, manufacturing complexity, and poor infiltration of engrafted cells, etc still, need to be overcome for further treatment with different forms of cancer. Recently, the antitumor activities of CAR‐T and TCR‐T cells have shown great improvement with the utilization of CRISPR/Cas9 gene editing technology. Thus, the genome editing system could be a powerful genetic tool to use for manipulating T cells and enhancing the efficacy of cell immunotherapy. This review focuses on pros and cons of various gene delivery methods, challenges, and safety issues of CRISPR/Cas9 gene editing application in T‐cell‐based immunotherapy.

## 1 INTRODUCTION

### 1.1 Cell immunotherapy and the challenges it faces

With precise targeting and impressive efficacy, CAR‐T (Chimeric Antigen Receptor Engineered T cell) and TCR‐T (T Cell Receptor Engineered T cell) cell therapies have become powerful and innovative therapeutic modalities for cancer patients. CARs are recombinant receptors that redirect the T‐cell activity towards target cells expressing specific surface antigen, independent of the classic peptide/MHC‐TCR recognition patterns. While TCR‐T cells are directed to recognize tumor‐specific peptide epitopes‐generated from inside the cells with the dependence on MHC molecules.

**Figure 1:**
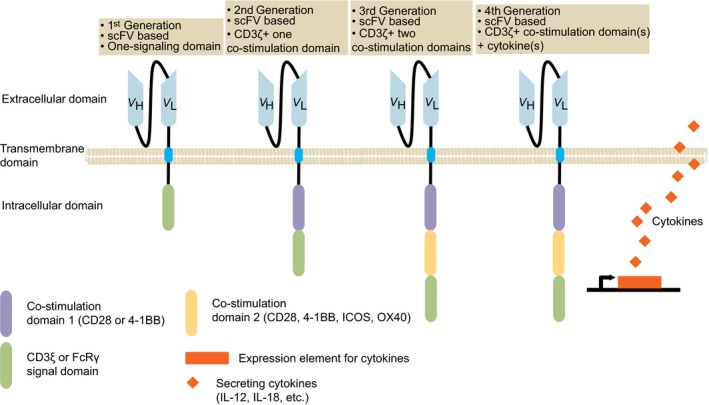
Different generations of CAR. The basic design of CAR is composed of an extracellular binding domain (usually a scFv), a hinge, transmembrane domain and one to three intracellular domains. The fourth‐generation‐CAR‐T cells are engineered to deliver a transgenic payload, such as proinflammatory cytokines, released upon engagement of CAR with its target. CAR, chimeric antigen receptor; scFv, single‐chain variable fragment

The first‐generation of CAR consists of the binding moiety from a monoclonal antibody fused to the constant regions of a TCR,^1^, ^2^ and later this design was modified to use a single‐chain Fv fragment (scFv) of an antibody linked with CD3 zeta or FcγRIIIA γ signaling chain (Figure 1).^3^, ^4^ Such engineered T cells specifically lysed target cells and produced cytokines.^5^, ^6^, ^7^, ^8^ However, clinical studies showed its limited antitumor efficacies (Table 1), probably owing to the short persistence of CAR‐T cells *in vivo*.^9^, ^10^ The second‐generation of CAR (Figure 1) provided a costimulatory signal in combination with the primary activation signal.^11^, ^12^ These CAR‐T cells have a higher level of cytokine production, improved persistence in vivo and potent clinical activities (Table 1),^13^, ^14^ enabling FDA's first two approvals of CAR‐T therapies in 2017. The third‐generation of CAR contained two costimulatory domains combined with an activation domain (Figure 1), which suggested an enhancement of antitumor response compared to the second‐generation CAR‐T cells (Table 1).^15^, ^16^ A clinical trial comparing the antitumor efficacy between the second‐ and the third‐generation CAR‐T cells is currently underway (NCT01853631). The fourth generation of CAR‐T cells (Table 1), engineered based on the backbone of the second‐generation CAR, were equipped with an inducible expression cassette to produce a transgenic cytokine, for example, IL‐12, IL‐18, upon the engagement of CAR to the specific tumor target (Figure 1).^17^

**Table 1:** Different generations of CAR‐T cells

Generation	First generation	Second generation	Third generation	Fourth generation
Composition of CAR	scFv, hinge, Fc receptor γ or CD3 ζ signaling chain	scFv, hinge, transmembrane region, one costimulatory domain, CD3 ζ	scFv, hinge, transmembrane region, two costimulatory domains, CD3 ζ	scFv, hinge, transmembrane region, one or two costimulatory domains, CD3 ζ, transgenic payload (cytokines, antibodies, enzymes, etc)
Preclinical studies	Effective in vitro and in mouse models	Superior antitumor effects, improved persistence, and higher level of cytokine production than first‐generation CAR	Compared to second‐generation CAR‐T, showed enhanced antitumor potency in some studies	Improved the effector function of CAR‐T cells in suppressive tumor microenvironment of solid tumor; provided a safety switch
Summary of clinical results	Limited antitumor efficacy due to short persistence in vivo	Most often used in clinic, significant antitumor response in various hematological malignancies	Used in a few clinical studies and most of them are still ongoing. Limited data suggested good antitumor efficacies and modest toxicity in B cell malignancies	Clinical studies for hematological malignancies and solid tumors are still in the early stage
Reference	1, 2, 3, 4, 5, 6, 7, 8, 9, 10	11, 12, 13, 14	15, 16	17

Abbreviations: CAR, chimeric antigen receptor; scFv, single‐chain variable fragment.

CAR‐T and TCR‐T cell therapies are showing promising results for cancer treatment, especially for targeting the B‐cell lineage‐restricted CD19 molecule expressed on B‐cell leukemias and lymphomas with CD19‐specific CAR‐T cells.^13^, ^18^, ^19^, ^20^ However, challenges including poor persistence, long manufacturing time, and limited infiltration of engineered T cells into immunosuppressive environment, still remain to be addressed. Through disrupting TCR and HLA genes, knocking out checkpoint inhibitory molecules, etc, CRISPR (Clustered Regularly Interspaced Short Palindromic Repeats)/Cas9 (CRISPR‐Associated protein) technology holds enormous promise to enhance T‐cell functionality and improve drug efficacy.

### 1.2 CRISPR‐Cas9 genome editing system: a genetic tool to enhance T‐cell functionality

Cas9 functions as a RNA‐dependent endonuclease and can be directed to the DNA target sites under the limitation of the protospacer adjacent motif (PAM), guided by a chimeric single‐guide RNA (sgRNA).^21^ Cas9/sgRNA system could target any DNA sequence of interest by changing sgRNA guide sequence, and cut DNA to cause double‐strand breaks (DSBs).^22^ These DSBs are repaired by error‐prone nonhomologous end joining (NHEJ) or precise homology‐directed repair (HDR) pathways. NHEJ leads to insertions or deletions (indel) of target gene and makes gene knock‐out possible. HDR uses assisted recombination of DNA donor templates to reconstruct cleaved DNA with precise repair, which could be used to knock‐in desired DNA.

Compared to other genome editing strategies such as transcription activator‐like effector nucleases (TALENs), and zinc‐finger nucleases (ZFNs), CRISPR/Cas9 genome editing is more rapid, cost‐effective, and it has been applied widely in plants and animals with its easier feasibility. Differences among the three types of genome editing systems have been discussed in numbers of review papers.^23^, ^24^, ^25^, ^26^

## 2 APPLICATIONS OF CRISPR/CAS9 TECHNOLOGY IN T‐CELL THERAPY

Although commercial products of CAR‐T have been successfully lunched,^20^, ^27^ including Kymriah and Yescarta from Novatis and Gilead/Kite, respectively, there is still much room to improve the existing T‐cell therapy. We summarized the recent progress about how CRISPR/Cas9 system could be harnessed to produce advanced CAR‐T cell products, with lower cost, reduced risk of causing malignancies, improved antitumor activities and efficacies (Table 2).

**Table 2:** Applications of CRISPR/Cas9 technology in T‐cell therapy

Application	Generate off‐the‐shelf CAR‐T	Knock‐in CAR or TCR	Knock‐out checkpoint molecules	Generation of CAR‐T cells expressing exogenous cytokines
Summary	TCR, B2M and PD‐1 molecules were eliminated simultaneously to enhance the antitumor activity. Other genes such as CTLA‐4 and Fas were also disrupted together with TCR and B2M	CAR or TCR cassette is knocked into endogenous TCR gene locus to mitigate GvHD	PD‐1, CTLA‐4, and LAG‐3 genes were knocked out separately or in combination	Beneficial cell cytokines (‐IL‐12, IL‐15, IL‐18, IL‐17, etc) can be knocked in designed gene locus
Advantages	Cheaper and faster, more potent	Avoid random integration; uniform CAR expression	Higher efficacy, less side effects, durable	More natural, less side effects
Disadvantages	The elimination of HLA‐class I could increase the attack from NK cells	Low knock‐in efficiency	Potential off‐target effects	Limited knock‐in efficiency
References	28, 29, 30, 31	33, 34	35, 36, 37, 40, 41, 42, 45	46, 47, 48, 49, 50

Abbreviations: CRISPR/Cas9, clustered, regularly interspaced, short palindromic repeats/ CRISPR‐associated protein 9. CAR, chimeric antigen receptor; TCR, T cell receptor; PD‐1, programmed cell death protein 1; CTLA‐4, cytotoxic T‐lymphocyte–associated antigen 4; GvHD, graft‐vs‐host disease; LAG‐3, lymphocyte activation gene 3; IL, interleukin.

### 2.1 Generation of off‐the‐shelf CAR‐T cells

**Figure 2:**
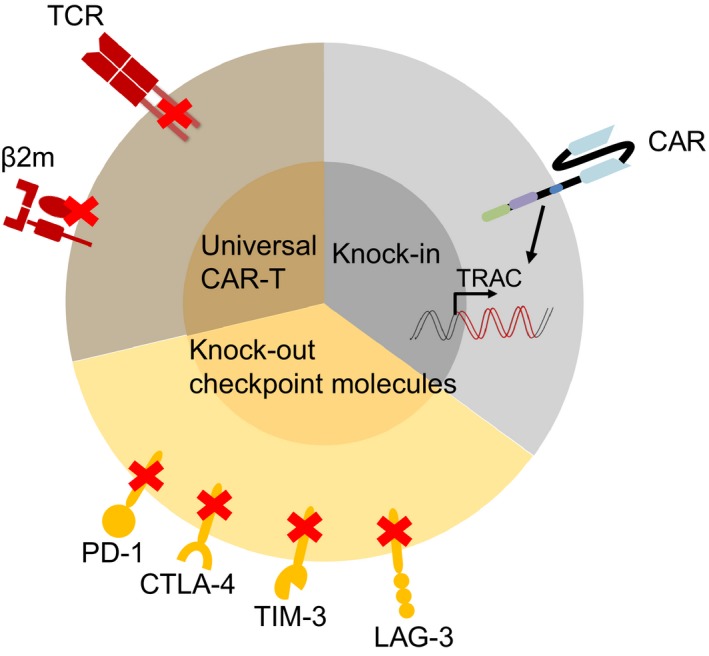
Applications of CRISPR/Cas9 technology in CAR‐T cell therapy. Pie diagram shows that three main aspects of CRISPR/Cas9 system can be applied in CAR‐T cell therapy: to generate universal CAR‐T cell products by disrupting endogenous TCR and MHC molecules, knock‐in CAR at a designed gene locus such as TCR locus to avoid random integration, and knock‐out checkpoint inhibitors to improve antitumor activities. CRISPR/Cas9, clustered regularly interspaced short palindromic repeats/ CRISPR‐associated protein 9; CAR, chimeric antigen receptor; TCR, T‐cell receptor

Current CAR‐T therapy mostly focused on autologous T cells owing to the limitation of intrinsic MHC restriction. To shorten the manufacture cycle and lower the cost of CAR‐T cell products, the concept of off‐the‐shelf CAR‐T cells was emerged (Figure 2). Endogenous TCR on allogeneic T cells were eliminated by ZFN and TALEN to avoid graft‐vs‐host disease (GVHD), and HLA molecules were disrupted to prevent a rejection from recipient's immune system.^28^, ^29^, ^30^ It has been reported that universal CAR‐T cells with TCRα chain and CD52 gene disrupted by TALEN were infused to two infants with relapsed refractory CD19^+ ^ALL,^31^ demonstrating the therapeutic potential of gene‐editing technology.

Compared to ZEN and TALEN technologies, CRISPR/Cas9 gene editing system holds greater promise due to its simplicity and high effectiveness to increase the efficacy of therapeutic agents or work as standalone therapeutics. *TRAC* and *B2M* genes have been knocked out simultaneously by CRISPR/Cas9 to generate universal CAR‐T cells.^32^ Besides, to improve antitumor activity, multiplex genomic editing of CAR‐T cells by CRISPR/Cas9 has been reported.^33^, ^34^ This one‐shot CRISPR system has shown to improve gene targeting efficiency and facilitate the manufacture of universal CAR‐T cells deficient in CD3 and HLA‐class I.^33^

Nevertheless, there is a potential issue when β2M and TCR gene loci are eliminated to prevent allo‐rejection. The elimination of HLA‐class I of T cell could increase the attack from NK cell due to its “missing self” phenotype,^35^ which should be taken into consideration for the future therapy.

### 2.2 Mitigation of malignancy risk by knocking in CAR or TCR at a designed gene locus

To avoid oncogenic transformation and transcriptional silencing caused by random integration of CAR into genome by lentivirus infection, knocking in CAR at designed gene locus via homologous recombination (HR) has been achieved (Figure 2). Schumann et al^36^ conducted a targeted nucleotide replacement in *CXCR4* and *PD‐1 (PDCD1)* gene loci by electroporating Cas9:sgRNA ribonucleoproteins (Cas9 RNPs) with homology‐directed repair template oligonucleotides, establishing applications of Cas9 RNP technology for genome engineering in human T cells. Eyquem et al showed that human T cells were electroporated with Cas9 mRNA and sgRNA to specifically insert a CD19‐specific CAR into TRAC locus, which resulted in not only uniform CAR expression but also enhanced T‐cell potency.^37^ These results indicate site‐specific knocking‐in a CAR may provide a safer and potent T‐cell product.

In addition to CAR knock‐in, a TCR that recognizes NY‐ESO‐1 tumor antigen has also been knocked into endogenous TCR gene locus, giving rise to specific recognition of tumor antigens and productive antitumor cell responses.^38^ Such precise knock‐in of CAR or TCR into a specific gene locus by CRISPR/Cas9 system leads to the enhancement of antitumor responses and brings additional clinical benefits to the patients engrafted with engineered T cells.

### 2.3 Knocking out of inhibitory checkpoint molecules to improve antitumor activity

To conquer the inhibitory effects of immune checkpoints in human T cells, and protect normal cells from being disrupted by checkpoint inhibitors in a nonspecific manner, PD‐1 gene in T cells has been abolished by CRISPR/Cas9 to enhance the cytotoxicity against tumor target cells (Figure 2).^39^, ^40^ Hu et al reported that PD‐1 gene was eliminated in anti‐CD133 CAR‐T cells by nucleofection of CRISPR/Cas9 plasmids,^41^ producing enhanced cytotoxicity of CAR‐T cells and inhibition of tumor growth. Interestingly, it has been reported that blockade of PD‐1, LAG‐3 (Lymphocyte Activation Gene 3) or CTLA‐4 led to a compensatory upregulation of the other checkpoint pathways,^42^ which means combinatorial blockade strategies should be applied in practice. This conclusion is in accordance with Tanvetyanon's review that combinatorial blockade of PD‐1 and CTLA‐4 may produce a higher antitumor response than PD‐1 blockade alone in patients.^43^ The clinical potential of combined disruption of PD‐1^44^, ^45^ and LAG‐3^46^ has also been explored in the CAR‐T cell therapy.

Except knock‐out of checkpoint molecules, an alternative way is to coexpress the PD‐1‐blocking scFv with CAR, which has improved the antitumor activities of CAR‐T cells.^47^, ^48^ CRISPR/Cas9 has also been used in TCR‐T cells to disrupt PD‐1 gene.^49^ The clinical trial with the strategy to disrupt endogenous TCR and PD‐1 gene is ongoing (NCT03399448).

### 2.4 Generation of CAR‐T cells expressing exogenous cytokines for efficacy improvement

In addition to TCR engagement (Signal 1) and costimulatory signaling (Signal 2), cytokines play essential roles in regulating T‐cell function. Constitutive expression of IL‐12 in CAR‐T cells to destroy antigen‐loss cancer cells,^50^ and improve antitumor efficiency^51^ has been reported. IL‐15, which is functionally associated with T‐cell memory, was coexpressed with anti‐CD19 CAR to develop long‐term persistence of CAR‐T cells.^52^ IL‐18 was also expressed in CAR‐T cells to augment antitumor effects against melanoma.^53^ IL‐7 and CCL19 have been expressed together with anti‐CD20 CAR‐T to treat preestablished solid tumors.^54^

Most of the cytokines discussed above including IL‐12, IL‐15, IL‐18, and IL‐7 are overexpressed through gamaretrovirus or lentivirus. Their expression is artificially regulated and may lead to side effects such as T‐cell exhaustion caused by higher secretion of cytokines. A better strategy would be to drive the expression of these cytokines under the control of an endogenous promoter through knocking in by CRISPR/Cas9 at a designated gene locus such as TRAC.

## 3 DIFFERENT METHODS OF GENE DELIVERY SYSTEMS FOR CRISPR/CAS9‐BASED CELL IMMUNOTHERAPY

**Figure 3:**
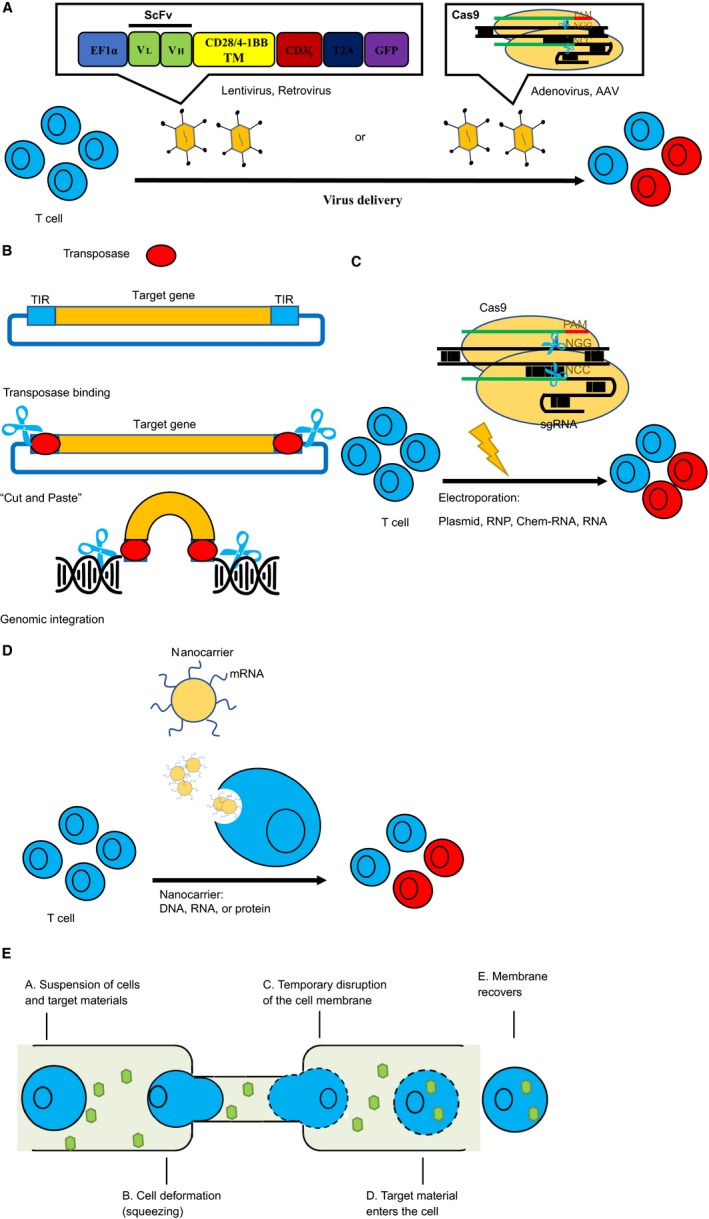
Different methods of delivery systems for CRISPR/Cas9 based cell immunotherapy. A, Viral delivery of CAR or Cas9/sgRNA into T cells. CAR genes are delivered by lentivirus or retrovirus and integrate into the genome for stable expression, while cas9 and sgRNA are often transiently expressed by AdV or AAV. B, Transposition mechanisms of DNA transposon. DNA transposon contains a target gene in the middle, flanked by TIRs. Transposase binds to the TIRs, and mobilizes the transposon for integration into the target genome via a cut‐and‐paste mechanism. TIR: terminal inverted repeats. C, Cas9 and sgRNA are delivered by electroporation in the form of plasmid, RNA, chem‐RNA, or RNP. D, Delivery of DNA, RNA, or protein into target cells via nanoparticles. E, Five main steps of delivery via CellSqueeze technology. scFv, single‐chain variable fragment; Cas9, CRISPR associated protein 9; AAV, adenovirus associated virus; RNP, ribonucleoprotein

An efficient gene delivery system is critical for the success of CAR and TCR cell therapies and the efficacy of gene editing. There are several major delivery systems (Table 3). Gamaretro‐, lenti‐virus‐based gene delivery, and transposon systems are used to stably express CAR/TCR in T cells, while adenoviruses (AdV), adeno‐associated viruses (AAV), electroporation and nanocarriers are used to deliver CRISPR/Cas9 for transient expression (Figure 3). A new delivery system called cell squeezing, recently emerges to deliver a wide range of compounds including DNA, RNA, and protein.^55^, ^56^, ^57^

**Table 3:** Different methods of delivery systems for CRISPR/Cas9‐based cell immunotherapy

		Forms of payload	Capacity	Advantages
Viral delivery system	Infection	AdV	7.5‐37 kb	1) Broad infectivity; 2) High titers; 3) Large cloning capacity; 4) Transient expression without integration
		AAV	5 kb	1) Relatively broad host spectrum; 2) Low immunogenicity; 3) Transient gene expression
		Gammaretroviral & lentiviral vectors	8‐9 kb	1) Stable gene expression; 2) Lentivirus infects dividing and nondividing cells
Nonviral delivery system	Electroporation	Minigene/minicircle (Transposon)	More than 100 kb	1) Lower immunogenicity; 2) Efficient stable genome modification; 3) Reduced cost
		DNA	More than 100 kb	1) Easy to operate; 2) Lower cost; 3) scalable manufacturing
		mRNA	Flexible	1) Higher efficiency; 2) Rapid expression; 3) Reduce off‐target effect
		RNP	Flexible	1) Lower off‐target; 2) Lower cellular toxicity
	Nano‐carrier	RNA, DNA, or protein	Flexible	1) Flexible payload sizes and formats; 2) Low immunogenicity; 3) Transient or stable gene expression
	Squeeze	Different bioactive materials, including small molecules, polysaccharides, siRNA, proteins, carbon nanotubes and quantum dots	Flexible	1) Diverse range of payloads; 2) Higher efficiently; 3) Unchanged expression profiles; 4) High throughput; 5) Improved safety

Disadvantages	Reference	Clinical trials
1) High immunogenicity	55, 56, 57	None
1) Limited cargo size; 2) Difficult to produce pure viral stock	58, 59	Projected by Editas Medicine in LCA10 patients
1) Genotoxicity; 2) Random integration	60, 61, 62	CAR‐T (gammaretroviral): NCT01583686, NCT02744287 (lentiviral): NCT02935543, NCT03274219, NCT03166878
1) Cellular toxicity (Transposon); 2) Random integration	63, 64, 65	NCT03389035, NCT00968760, NCT01497184, NCT01362452, NCT01653717, NCT02529813
1) Reduced cell viability; 2) Spontaneous vector integration	28, 29, 37, 40	CRISPR: NCT03057912, NCT03164135, NCT03655678, NCT03545815, NCT03081715, NCT03398967, NCT03690011, NCT02863913, NCT02867345, NCT02867332, NCT02793856, NCT03044743
1) Medium cost; 2) Fast degradation		NCT03399448, NCT03166878
1) High cost; 2) Fast degradation		None for CRISPR/Cas9
Requires special expertise	66, 67, 68, 69, 70	NCT02340156, NCT03020017, NCT01455389
Requires special facilities	51, 52, 53, 71	None

AdV, adenovirus; AAV, adenovirus associated virus; RNP, ribonucleoprotein.

LCA10, TYPE 10 leber's congenital amaurosis; CAR, chimeric antigen receptor; CRISPR, clustered, regularly interspaced, short palindromic repeats.

### 3.1 Virus‐mediated gene delivery

Most commonly used viral vectors are derived from AdV, AAV, gammaretrovirus or lentivirus (Figure 3A).^58^ AdV‐derived vectors are able to infect a broad range of nondividing or dividing vertebrate cells and have many advantages (Table 3).^59^ Using AdV‐based CRISPR/cas9 system, Cheng et al demonstrated that gene editing in the mouse liver was highly efficient, specific, and persisted long in vivo while the expression of Cas9 protein was transient.^60^ Nevertheless, AdV has strong immunogenicity in nature, which raises safety concerns for clinical uses.^61^ AAV vectors have broad spectrum of target cell types and low immunogenicity, with its capacity less than 4.8 kb (Table 3). Using a dual‐vector system with SpCas9 (~4.1 kb) and sgRNA expressing separately, Swiech et al demonstrated effective editing of single or multiple genes in the mouse brain.^62^ On the other hand, Ran et al identified that smaller Cas9 ortholog, SaCas9 (~3.3 kb) with similar efficiency as SpCas9 allowed the delivery of SaCas9 and its sgRNA in a single AAV vector.^63^

Gammaretrovirus and lentivirus belong to the retroviral family and possess the intrinsic capability to integrate into the host genome, allowing long‐term and stable transgene expression (Table 3). Lentiviruses are able to infect both dividing and nondividing cells, while gammaretroviruses have been shown to preferentially infect dividing cells.^64^ CAR‐T cells were generated with 50%‐80% transduction efficiencies using replication‐deficient gammaretroviral or lentiviral vectors,^65^, ^66^ which are the main gene delivery systems in manufacturing CAR‐T cells for clinical usage. A major concern for retroviral and lentiviral vectors is the random insertion of transgene into chromosomes, posing risks of oncogenesis, although no apparent oncogenic consequences have been observed from existing clinical practice yet.

### 3.2 Transposon

Besides the viral vector delivery systems described above, Transposon has emerged as a new potential delivery tool for transferring genes of interest (Figure 3B). The vector system of DNA transposon comprises a transposon containing a gene of interest flanked by terminal inverted repeats (TIRs), and a transposase that binds to TIRs.^67^ These nonviral vector integration systems, such as *PiggyBac* (PB) and *Sleeping Beauty* (SB), also showed advantages for gene delivery (Table 3).

Transposon system was exploited in clinical trials for cancer immunotherapy. Human T cells genetically modified by the SB transposon/transposase system to express a CD19‐specific CAR were evaluated in 2016.^68^ The clinical results demonstrated that these SB platform‐engineered CAR‐T cells were safe, and further supported the clinical development of this nonviral gene therapy approach. Clinical trials at MD Anderson Cancer (Table 3) have shown that these transposon‐engineered CAR‐T products work robustly and feasibly.^69^

### 3.3 Electroporation

Cas9 mRNAs or proteins were electroporated into CAR‐T cells (Figure 3C) to knock‐out specific genes like TCR,^33^ CTLA‐4, and PD‐1 gene.^32^, ^33^, ^44^ Ren et al accomplished a versatile system for rapidly generating multiplex genome‐edited CAR‐T cells, by lentiviral infection of one‐shot CAR vector with multiple sgRNAs and electroporation of Cas9 mRNA.^33^ Other labs developed a protocol for combined Cas9 RNP‐mediated gene editing and lentiviral transduction to generate PD‐1 deficient anti‐CD19 CAR T cells.^44^ Hu et al explored a simplified protocol for generating PD‐1 deficient CD133‐specific CAR T cells, by nucleofecting plasmids of CRISPR/Cas9 system to disrupt PD‐1 gene and piggyBac transposon system for CAR gene expression. This convenient method avoids manufacturing of RNAs or proteins and reduces the processing of T‐cell modification.^41^

### 3.4 Nanocarriers

Nanoparticles are emerging synthetic delivery systems with favorable characteristics (Table 3).^70^, ^71^ The flexible design of nanoparticles allows to carry versatile types of cargoes or a combination of multiple components (Figure 3D). A novel nanocarrier CRISPR‐gold, designed to deliver three components simultaneously, was able to internalize by primary cells and stem cells via endocytosis.^72^ Moreover, administration of CRISPR‐gold carrying Cas9 RNP and donor DNA to correct the *Dystrophin* gene mutation in MDX mice, resulted in the gene repair and restored the protein expression.

Selective targeting of specific cell subgroups is another feature that can be achieved via nanocarriers. In the application of Stephan lab, plasmids encoding CD19‐targeted CAR gene flanked with piggyBac system were coencapsulated in such nanoparticles conjugated with CD3‐targeting antibodies. The strategy showed robust CAR production inside the T cells both in vitro and in vivo.^73^ This system could also favor transient expression of transgenes by delivering mRNA, as Moffett et al described as “hit‐and‐run programming.”^74^

The key benefit of using nanoparticle over electroporation is high viability and expansion capability of manipulated cells, allowing its broader applications.

### 3.5 Microfluidics‐based CellSqueeze

Although gene delivery by electroporation has been wide‐used, it was found by genome‐wide approach that electroporation treatment may disrupt the expression profiles of key functional transcripts and lead to the perturbation of cytokine secretion. A microfluidic delivery system called cell squeezing was recently used for compound delivery. Its mechanism of action is based on mechanical membrane disruption (Figure 3E), which has minimal effects on transcriptional responses and will not modulate T‐cell activity.^75^ The CellSqueeze technology from SQZ Biotechnologies Co. (Table 3) is able to introduce a wide range of compounds into varieties of cell types like immune cells, embryonic stem cells etc.^55^, ^56^, ^57^

Compared with other delivery systems described above, CellSqueeze technology could achieve high delivery efficacy without adversely affecting cell viability and expression profiles.

## 4 CHALLENGES AND THE FUTURE DIRECTIONS OF THE APPLICATION OF CRISPR/CAS9 TECHNOLOGY

### 4.1 An increased risk of tumor malignancy

CRISPR‐Cas9 technology has made gene editing simpler and faster than ever. However, a study points out this popular gene‐editing tool could inadvertently cause cancer. It found that Cas9 RNP delivery triggers a p53‐dependent DNA damage response that suppresses gene correction.^76^

### 4.2 Failure of genome editing caused by the immunogenicity elicited from anti‐Cas9 responses

A recent study showed that the most widely used forms of CRISPR could be an immunogen in humans. Instead of modifying the genome while used therapeutically, the editing tool could trigger an adaptive immunity to Cas9 proteins, raising considerable concerns for the future CRISPR clinical trials.^77^, ^78^

### 4.3 An increased risk of off‐target mutagenesis

The off‐target mutation, which may cause genomic instability and disrupt the functionality of other normal genes, is still the major concern of CRISPR/Cas9 system in biomedical and clinical application. Although the targeting specificity of Cas9 is believed to be tightly controlled by the guide sequence of sgRNA and the presence of PAM, potential off‐target cleavage activity could still occur with even three to five base pair mismatches in the PAM‐distal part of the sgRNA‐guiding sequence.^79^ Moreover, Cas9 stays in the cells for a period of time after treatment, which increases the incidence of DNA being cut in the wrong place.

### 4.4 Inefficient delivery systems for CRISPR/Cas9

There are several delivery systems for CRISPR/Cas9 as described previously, but the delivery efficiency of each system is still not satisfying, especially for in vivo application. In addition, large DNA fragment knock‐in and multiplex genome editing are generally hard to achieve.

## 5 CONCLUSIONS AND FUTURE DIRECTIONS

CRISPR/Cas9 has provided a simple, cheap, and fast way to manipulate genomes. CRISPR‐edited CAR‐T and TCR‐T cells hold out great hope and potential for the next generation cancer immunotherapy, particularly for the treatment of solid tumors. Considering the safety issues related to CRISPR/Cas9 system, mutated Cas9 including Cas9 nickase,^80^ truncated sgRNA,^81^ or other more accurate nucleases with longer PAM sequences are explored to reduce off‐target effect. As to improve delivery efficiency of CRISPR, new delivery platforms like nanocarrier and CellSqueeze technology, have recently emerged. With rapid improvement in the field of gene therapy, CRISPR/Cas9 genome editing system is expected to have broader therapeutic applications in cancer immunotherapies.
